# Metal Oxide Nanomaterials: From Fundamentals to Applications

**DOI:** 10.3390/nano12234340

**Published:** 2022-12-06

**Authors:** Yuanbing Mao, Santosh K. Gupta

**Affiliations:** 1 Department of Chemistry, Illinois Institute of Technology, 3101 South Dearborn Street, Chicago, IL 60616, USA; 2 Radiochemistry Division, Bhabha Atomic Research Centre, Trombay, Mumbai 400085, India; 3 Homi Bhabha National Institute, Anushaktinagar, Mumbai 400094, India

This Special Issue of *Nanomaterials*, “Metal Oxide Nanomaterials: From Fundamentals to Applications”, highlights the development and understanding of different types of metal oxide nanoparticles and their use for applications in luminescence, photocatalysis, water–oil separation, optoelectronics, gas sensors, energy-saving smart windows, etc. The wide variety of applications covered by the 10 articles published here is proof of the growing attention that the use of metal oxide nanomaterials has received in recent years. Here, nanomaterials are defined, based on the October 2011 European Commission’s definition, as materials with one or more external dimension in the range of 1–100 nm. As the surface area per mass of a material increases, a greater proportion of the material can come into contact with the surrounding materials, thus affecting reactivity [1,2]. Nanomaterials have indeed revolutionized the world due to their unique properties and growing applications in all spheres of humankind, encompassing energy, health, and the environment.

Within this Special Issue, Gupta et al. designed and demonstrated excitation energy tunable light emission from barium zirconium oxide crystals as color-tunable phosphors [3]. The authors tuned the emission light from BaZrO_3_:Eu^3+^ crystals from orange to red based on the charge transfer and *f*-*f* transition excitation of an Eu^3+^ dopant, which is dictated by its magnetic and electric dipole transition probabilities. Enesca et al. showed the potential of Cu_2_O/SnO_2_/WO_3_ heterostructure powder in the efficient removal of pesticides photo-catalytically [4]. The photocatalytic mechanism corresponds to a charge transfer based on this three-component structure, where Cu_2_O exhibited a reduction potential responsible for O_2_ production and WO_3_ had an oxidation potential responsible for OH· generation. Liang and his colleagues successfully decorated TiO_2_ nanorods with a copper oxide layer through sputtering and post-annealing, which resulted in improved light absorption and photo-induced charge separations [5]. This led the composite nanorods to have enhanced photoactivity compared to the pristine TiO_2_ nanorods.

Other fascinating properties of nanocomposite based on metal oxides nanoparticles, particularly from ZnO, were explored by several other research groups [6]. For example, Si et al. synthesized Fe_3_O_4_@ZnO nanocomposites (NCs) to improve the stability of the viscoelastic surfactant (VES) fracturing fluid [7]. At a loading of 0.1 wt.%, this NC-VES nanocomposite showed superior stability at 95 °C or at a high shear rate and good sand-carrying performance and gel-breaking properties. Designing a surface with special wettability is an important approach to improving the separation efficiency of oil and water. Liu and co-authors demonstrated superhydrophobicity in both oil and water from their stainless-steel metal fibers coated with sol–gel-derived ZnO nano-pillars [8]. They found that their ZnO-coated stainless-steel metal fibers had a static underwater oil contact angle of 151.4° ± 0.8° and an underoil water contact angle of 152.7° ± 0.6° and was a highly promising candidate for both water-in-oil and oil-in-water separation in the industry. Maevskaya et al. studied the effect of Cu_2_O on the photo-induced alteration of the hydrophilicity of TiO_2_ and ZnO surfaces [9]. The Cu_2_O/TiO_2_ and Cu_2_O/ZnO heterostructures showed photo-induced decay of the surface hydrophilicity caused by both UV and visible light irradiation. Simeonov and his group carried out defect engineering of ZnO by nitrogen doping [10]. They demonstrated that nitrogen doping in ZnO led to an abundance of oxygen and zinc vacancies and interstitials and contributed to enhanced electron transport properties in ZnO:N films. As another example demonstrating advancements in the roles of structure and morphology in material properties, Naszályi Nagy et al. prepared silica NPs with a diameter of 50 nm and covered them with a monoclinic/cubic zirconia shell using a green, cheap, and up-scalable sol–gel method [11]. They confirmed that these silica@zirconia core@shell NPs bind as muchas 207 mg of deoxynucleoside monophosphates on 1 g of this nanocarrier at neutral physiological pH while maintaining good colloidal stability.

Two review articles are also included in this Special Issues. In the first review, Li et al. comprehensively discussed the development of two-dimensional (2D) nanomaterials with metal oxide nanoparticles for gas sensing applications [12]. They further emphasized recent advances in the fabrication of gas sensors based on metal oxides, 2D nanomaterials, and 2D material/metal oxide composites with highly sensitive and selective functions. In the second review article, Kim et al. presented recent advances in fabricating flexible thermochromic VO_2_(M) thin films using vacuum deposition methods and solution-based processes and discussed their optical properties for potential applications in energy-saving smart windows and several other emerging technologies [13].

In summary, this Special Issue presents just the tip of the iceberg of the broad, dynamic, and active fundamental research and applications in the developing field of metal oxide nanomaterials by collecting a few examples of the latest advancements. We hope that the readers enjoy reading these articles and find them useful for their research.
